# 3D Printing Technology for Smart Clothing: A Topic Review

**DOI:** 10.3390/ma15207391

**Published:** 2022-10-21

**Authors:** Shuangqing Wu, Taotao Zeng, Zhenhua Liu, Guozhi Ma, Zhengyu Xiong, Lin Zuo, Zeyan Zhou

**Affiliations:** 1 College of Engineering and Design, Hunan Normal University, Changsha 410081, China; 2 School of Materials Science and Engineering, Hunan University, Changsha 410082, China

**Keywords:** 3D printing, clothing, traditional dress, smart materials

## Abstract

Clothing is considered to be an important element of human social activities. With the increasing maturity of 3D printing technology, functional 3D printing technology can realize the perfect combination of clothing and electronic devices while helping smart clothing to achieve specific functions. Furthermore, the application of functional 3D printing technology in clothing not only provides people with the most comfortable and convenient wearing experience, but also completely subverts consumers’ perception of traditional clothing. This paper introduced the progress of the application of 3D printing from the aspect of traditional clothing and smart clothing through two mature 3D printing technologies normally used in the field of clothing, and summarized the challenges and prospects of 3D printing technology in the field of smart clothing. Finally, according to the analysis of the gap between 3D-printed clothing and traditionally made clothing due to the material limitations, this paper predicted that the rise in intelligent materials will provide a new prospect for the development of 3D-printed clothing. This paper will provide some references for the application research of 3D printing in the field of smart clothing.

## 1. Introduction and Motivation

Clothing is regarded as an important human social activity. At present, human beings not only pursue the beauty and comfort of clothing, but also pay attention to the expression of comfort and personality, which poses a new challenge to the clothing industry. The “smart clothing” made through the combination of traditional clothing and intelligent technology has unlimited market prospects because of its incomparable advantages over traditional clothing, and the outbreak of related industries is just around the corner. This so-called intelligent clothing refers to the introduction of sensing technology, microelectronic technology and information technology into people’s daily clothing, such as for health monitoring [1,2], energy collection [3], intelligent sensing [4], displaying information [5], data transmission and other modules [6,7]. Because of its unique preparation technology and product characteristics, 3D printing and wearable technology have experienced an explosion in popularity in the last couple of years in fashion disciplines from both industry and academia. This 3D printing technology is a great invention of the 20th century, which is called the iconic technology of the third industrial revolution. The advantage of 3D printing technology is particularly obvious in the clothing industry [8]. Since the first 3D-printed dress came out [5], it opened the door for new trends. Many well-known designers have applied 3D printing technology into clothing to design clothing with the science and technology of the future, which has brought unlimited possibilities to the clothing industry. Additionally, the application of 3D printing technology completely subverts consumers’ cognition of traditional clothing.

**Figure 1:**
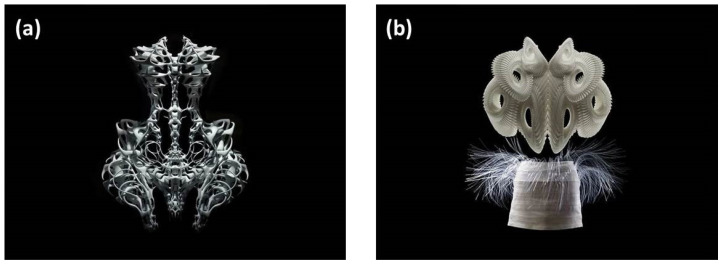
Iris van Herpen’s 3D-printed clothing from 2010 (**a**) and 2011 (**b**) in high-definition series [24].

As an epoch-making technology, 3D printing plays a vital role in different fields, from the F-35 airplane [9] to the Urbee 2 automobile [10], and from surgical organ substitutes [11] to food printers [12]. Meanwhile, the application of 3D printing technology has also brought more inspiration and creativity to clothing designers. Compared with traditional clothing production, 3D printing technology can provide personalized customization by scanning the wearer’s body parts before printing [13,14]. This personalized customization ability of 3D printing technology has attracted much attention in the field of clothing. In fact, the research and applications of 3D printing in clothing at home and abroad are numerous. As the leaders of sportswear brands, Nike, Under Armour, Adidas and other companies lead the trends in the shoe market, for example, by making their products via 3D printing technology and emphasizing personalized customization. For example, Nike [15,16] made a pair of shoes for American national athlete Allyson Felix by using 3D printing technology. This technology shortens the cycle of shoe production from three months to a few hours, which makes it convenient for athletes to adjust the comfort of their shoes. Therefore, athletes can have the most suitable shoes in time. Subsequently, Under Armour [17,18,19] launched a 3D-printed sneaker called the “Archi-TechFuturist”. Compared with the original 3D-printed sneaker from Nike, it has made a change in the 3D-printed sole, which can provide better cushioning and more stable support for athletes. However, the product, as a limited edition, has not been mass-produced thus far. The Adidas Group [20,21] launched a corresponding 3D-printed shoe named the “3D Runner Pump”. Meanwhile, the team from Reebok [22] developed a pair of 3D-printed shoes called the “Reebok Liquid Speed”, which has unique numbers and is limited to 300 pairs. Recently, Chinese innovative footwear brands SCRY Lab and Heliot Emil designed a pair of 3D-printed shoes and displayed them at Paris International Fashion Week 2022. Additionally, Chinese well-known casual sports shoe brands Li Ning, Peak and Hongxing Erke have also launched 3D-printed shoes and achieved certain research results. The application of 3D printing in the clothing field attracted the attention of high-end designers in the fashion world and also caused a lot of creative inspiration. Iris Van Herpen [23] was the first fashion designer to put 3D-printed clothing on the fashion stage. Iris van Herpen is a young and talented female designer, who is especially good at designing according to the material of the clothing itself. She has worked with Alexander McQueen and Viktor & Rolf, and her design style has been influenced by these two major brands. The avantgarde and creative appearance of clothes makes Iris van Herpen’s works [24] (Figure 1) visually impactful and eye-catching. Functional 3D printing technology can realize the perfect combination of clothing and electronic devices. The combination has provided people with the most comfortable and convenient wearing experience and promoted smart clothing to realize specific functions. From the “fantasy” of the Space S series in 2011 to the “miniature” series in 2012, and from the 2013 clothing theme of “voltage” to the fashion show of the “hypnotic clothing” of the Fwhite W series in 2019, these were all conceptual clothing made using 3D printing technology. Due to their special formability and artistic expression, these clothes were once recognized as the leading experimental designs [25,26,27]. In 2012, the world’s first 3D-printed bikini, designed by fashion designer Mary Huang and 3D-modeling expert Jenna Fizel, also went on sale [28]. The swimsuit was printed by laser sintering and then seamlessly spliced. The swimsuit was printed from nylon, which is so wear-resistant and flexible that even thinner swimsuits do not have to worry about damage. The Chanel brand also launched 10 suits made by 3D printing technology, while the jewelry accessories were also 3D printed. Not long ago, the autumn and winter collection “Future Elf” (the Spirit of the Future), launched at Paris Fashion Week, was also a masterpiece of 3D printing. Subsequently, more and more fashion industries have introduced 3D printing technology into their products. The representative products are as follows: the suit and tie from Japanese design studio Monocircus, the DNA 3D-printed shoes designed by American designer Pensar and the 3D-printed hat by MGX in Belgium [29]. All of them represent the coexistence of beauty and technology.

In addition, 3D printing has come to the fore in the commercialization of clothing. For example, the well-known Shapeways website, where customers can select templates uploaded by designers and place orders, provides the services of 3D printing and mailing of clothing products. The company is committed to printing one piece of clothing. In fact, Victoria’s Secret has worked with the site to print products. At the 3D Printshow in London in 2015, the company launched a 3D-printed dress which was printed into a ball and then unfolded with a greater variety of colors by using the ductility of the material. These cases will promote the wide application of 3D-printed clothing.

Due to the limitations of raw materials, 3D-printed clothing has a certain gap in terms of flexibility and comfort compared to traditional clothing. However, with the continuous research and development of materials, there has been much progress achieved in the field of creating flexible fabric structures and developing functional clothing, as well as in the field of electronic textiles. In addition, the combination of smart materials and 3D printing technology will provide more opportunities and challenges for the clothing field which will promote the development of smart clothing. Although 3D printing has been applied in the clothing field and made some achievements, many scientific issues around the raw materials used in the 3D process and around 3D technology used in smart clothing need to be solved. Additionally, the related literature about the application of 3D printing in the clothing field is scattered. Thus, in order to promote the development of 3D printing in the clothing field, this paper will give a review of the progress of the applications of 3D printing in traditional clothing and smart clothing through two mature 3D printing technologies normally used in the field of clothing.

## 2. The Method of 3D Printing in Clothing

Three-dimensional printing technology, increases the spatial dimension based on two-dimensional printing, which makes the inkjet printing pattern have a three-dimensional height. Different from the traditional material-reduction processing technology (such as turning, milling, grinding and so on), 3D printing technology uses a layer-by-layer superimposed material-forming method. The basic principle is the following procedure: based on the digital computer-aided design (CAD) model, the three-dimensional model is sliced by layered technology to obtain the print path data of each layer, and then, the bindable materials such as powdered metal or plastic are superimposed layer by layer through the printer and bonded together. Generally speaking, 3D printing products need to go through the following four steps, namely, 3D modeling, layered slicing, printing and post-processing [30]. Thus far, there are dozens of types of 3D printing technology [31]. However, in the field of 3D-printed clothing, two kinds of forming technologies are mainly used, namely, fused deposition modeling (FDM) and selective laser sintering (SLS).

### 2.1. Fused Deposition Modeling (FDM)

The FDM process [32] uses high-temperature heated nozzles to melt and deposit materials layer by layer. The working temperature of common FDM printing equipment is about 300 °C, which means that the printing material not only has excellent thermoplastic processability, but also needs to have a lower molding temperature and certain solution strength. In addition, good adhesion is needed when bonding between layers to avoid cracking caused by thermal stress. As the most commonly used molding process, its main advantage is low-cost processing. However, this process can only print clothing with a simple shape based on the textile structure. Melnikova [33] pointed out that the FDM process can possibly form complex textile structures with the help of support structures. That is to say, it requires additional material consumption and time to deposit and print the fine support structure, and to remove the support in the post-processing stage. This not only increases the complexity of the molding process, but also limits the success rate of FDM molding printing due to the fine-structure printing [34].

### 2.2. Selective Laser Sintering (SLS)

The SLS process [35], firstly, preheats the powder material to below the melting-point temperature and, then, tiles the material. Subsequently, the controllable laser beam selectively carries out layer-by-layer sintering according to the layered cross-section during the slicing, and then, the finished product can be obtained by removing the excess powder after all the sintering of the tiled material is completed. Beecroft [36] explored the process of printing nylon powder by SLS to create flexible textile structures. It was found that the technology can print fabric structures with complex geometry without adding additional supporting structures during the process of forming clothing textiles. This shows that the SLS process is an ideal method for forming fine-structure textiles.

## 3. The Research Progress on 3D Printing in Clothing

Three-dimensional printing technology can realize the application of high-performance materials with different functions into clothing and accessories to build the core components of intelligent clothing [37,38,39,40,41,42,43,44]. The progress of the applications of 3D printing in clothing is introduced from the aspect of traditional clothing and smart clothing in this section.

### 3.1. The Progress of 3D Printing in Traditional Clothing

#### 3.1.1. The Basic Application of 3D Printing in Traditional Clothing

With the development of 3D printing technology, 3D printing has gradually been applied in clothing, textiles and other disciplines. Additionally, research on the materials in the field of 3D-printed clothing has also made preliminary progress [45]. Cakar [46] measured and analyzed the printed samples made from polylactic acid (PLA (soft)), PLA+ (hard) and Filaflex (TPE, namely, “Thermoplastic Elastomer”). The results show that Filaflex is the best material for the process of 3D printing. In addition, the study shows that the air cavity produced during the printing process can be reduced by optimizing the printing parameters, whereas the effect of residual stress on Filaflex materials can be controlled by adjusting the printing load. Kim [47] compared the difference between TPU (thermoplastic polyurethane elastomers) and ABS (acrylonitrile butadiene styrene) by combining traditional textile fabrics with 3D-printed materials. It is pointed out that the solid material of ABS can provide a high-quality output. However, due to the high surface roughness, the products need post-processing. Besides, ABS is more suitable for the production of a circular structure in clothing products. Meanwhile, flexible TPU provides a relatively smooth surface. Additionally, due to the intrinsic characteristics of this material, it is more suitable for connection patterns in clothing products, such as making hinge structures. Spahiu [8] printed a dress with arrows as its geometric structure using the FDM process. Fixed belts were added on both sides of the dress to make it easier to wear, and the whole dress was made of Filaflex flexible filament material for better flexibility. However, there is still a certain gap between the properties of printed materials and traditional textile materials. Yarwindran [48] carried out tensile and bending experiments on the Filaflex flexible filament and TPU flexible filament, respectively. The research showed that Filaflex is the most suitable and effective material for foot orthopedic insoles at present, as the TPU material will change its elastic properties due to heating during the printing process, and the bonding effect between layers is also not good.

In summary, the common materials used in the 3D-printed clothing are lighter plastics (ABS, PLA, nylon powder, etc.) and TPU, which is between plastics and rubber. ABS is a kind of hard plastic material with a low price. Therefore, it is more suitable for creative decorative contours of a large area in clothing technology. However, due to the low precision of the molding surface, it needs a post-treatment to obtain a fine surface. PLA, as a biodegradable and environmentally friendly biomaterial, means that the printed clothing using this material can be recycled in the event of damage or obsolescence. In addition, PLA is regarded as the most valuable polymer in the field of clothing because of its excellent stretching and melting properties. However, the clothing printed from PLA has high brittleness, and poor thermal stability and toughness with a lower impact strength than that of the ABS material. Therefore, it is not suitable for thin-walled clothing. Due to the high wear resistance and elasticity, flexible polyurethane (PU) [49,50] is mainly used to print sports shoes, such as midsoles, insoles and so on. Although the clothing printed with this material is not easy to deform, the material is expensive, and there are some uncontrollable changes from the original design after printout. In addition, as the printing is mainly limited by the viscosity of the material, when the temperature of the extruder decreases, it will adhere to the nozzle, which easily leads to blockage. Comparatively speaking, nylon has excellent tensile strength, elasticity and toughness. Furthermore, its output accuracy is better than that of ABS and PLA. However, the lower thermal deformation temperature leads to thermal shrinkage during the use of the product, which still cannot meet clothing requirements.

#### 3.1.2. The Flexible Structure of 3D Printing in Traditional Clothing

Most of the clothes printed with traditional materials are stiff, lack flexibility and are less comfortable; thus, they are mainly used for the display of conceptual clothing and cannot be used in the daily life of the public. However, a variety of flexible structural fabrics currently developed can improve 3D-printed clothing [51,52]. Melnikova [33] printed textile-based structures by using SLS and FDM technology. The results show that the brittle ABS cannot meet the fine-structure requirements, whereas the polylactic acid and nylon used in SLS may also be too hard for the application of typical textiles. However, soft polylactic acid, combined with less-flexible materials such as BendLay, has been shown to replicate some textile-based structures. These materials can develop new patterns and construction for clothing and be used in related fields. Thus, new designs and functions can be obtained, which cannot be realized by traditional textile fabrics. Bee croft [36,53] and others studied the tubular form of textiles based on 3D printing, which is realized by using the main structure of knitting. Based on the previous studies on the structure of 3D-printed fabrics, they explored the use of 3D-printed nylon powder to create a flexible weft-knitted structure. The results show the potential to print flexible, tubular textile-based structures at various scales that exhibit the properties of traditional knitted textile structures along with the mechanical properties of the material. In addition, these structures are printed in different thicknesses, demonstrating good flexibility, strength and tensile mechanical properties, which may make them viable solutions for use in the technical textile industry. The further study of different types of powder materials shows TPU may result in softer fabrics that may be more suitable for fashion applications.

Recently, researchers have developed 3D-printed chain-structure fabrics that can be bent and folded like traditional clothing fabrics [54]. Bloomfield [55] developed a Modeclix chain structure for the printing of nylon powder by the SLS process. Modeclix has been successfully used to create a range of clothing and fashion accessories to demonstrate the versatility of the link system. Sheets of connected textiles are printed with polyamide (nylon PA12). The material can be post-processed and then dyed into various colors, which causes the patterns and designs to be incorporated into textile forms. Since those textiles are made up of interchangeable links, it is a simple process to repair and reuse textiles. Modeclix is a designed system that integrates the factors of a circular economy into the product life cycle. That is to say, a piece of clothing can be reused to make new clothing, accessories, toys or other undiscovered products. Further development of the potential of the system through new components will also ensure the adaptability and future availability of textiles. The new advanced manufacturing parts can prolong and improve the service life of textiles, which will expand the field of application.

### 3.2. The Progress of 3D Printing in Smart Clothing

As a new product of the integration of electronic technology and wearable technology, smart clothing aims to achieve data collection and personalized displays in a comfortable and convenient way, which provides a new wearing experience for human beings. In the era of the mobile Internet, especially with the rapid development of cloud computing and the big data era, smart clothing will be able to achieve real-time data acquisition, remote processing and instruction issuance, which effectively will improve the use of existing clothing. Generally speaking, the field of smart clothing is in the early exploration stage of the outbreak of the industry. The development and innovation of conductive fabric materials, sensors, circuit technology and cloud computing, as well as big-data-related technologies, will provide a strong driving force for the development of the smart clothing market. Especially due to the continuous innovation and breakthrough of 3D printing technology and its functional materials, the comfort of smart clothing will be consistent with normal clothing. In the future, smart clothing will also form an intelligent system framework with smart families and smart cars, which can be used as personal life assistants to provide entertainment services, health maintenance, man–machine exchanges and other services.

**Figure 2:**
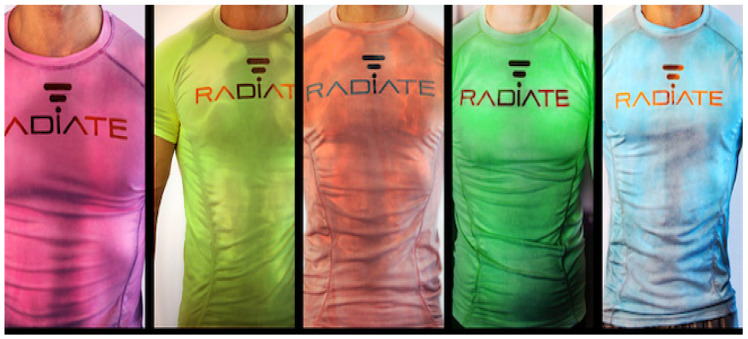
The Radiate sports T-shirt with various colors [67].

Flexible sensors with various functions, as an important part of smart clothing, can measure various motion states of the human body, such as acceleration, muscle extension and foot pressure [56,57,58,59,60,61,62,63]. They can also measure parameters related to the surrounding environment, such as location coordinates, temperature, humidity and atmospheric pressure. These flexible sensors with different functions and shapes provide an important tool for solving the problems of sensing measurements in health, medical, sports, industrial and military fields [64,65,66]. For example, Figure 2 shows a sports T-shirt [67] that can help athletes monitor the training of specific parts of the body or muscle groups. It can instantly sense the heat emitted from various parts of the body and transmit information through color changes. Thus, the athletes can keep abreast of the exercise of their muscles and blood vessels.

**Figure 3:**
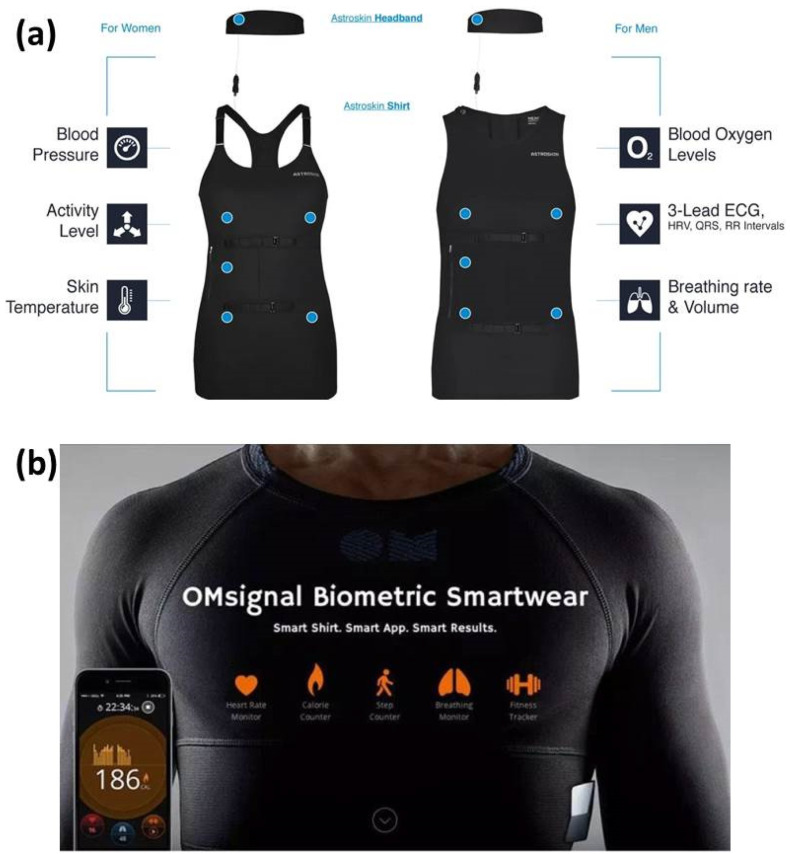
(**a**) An integrated biosensor in the exercise shirt. (**b**) The smart T-shirt with a built-in sensor [68,69].

An integrated biosensor (Figure 3a) in the exercise shirt developed by Hexoskin [68] measures heart rate, heart rate change and recovery, steps, calorie consumption and breathing data while tracking sleep and the environment at night, including sleep posture, heartbeat and breathing activity. Similarly, the smart T-shirt (Figure 3b) made by OMsignal [69] has a built-in sensor. In addition to monitoring routine data such as heart rate, breathing volume and calorie consumption, it also has novel functions such as removing moisture and promoting blood circulation. Furthermore, it is worth noting that its Bluetooth module can last 2–3 days at a time.

**Figure 4:**
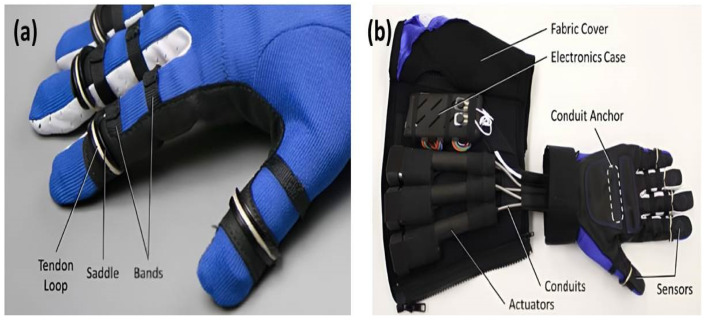
(**a**) A mechanical glove, Robo-Glove, with pressure sensors and other sensors jointly developed by General Motors and NASA. (**b**) The back and details of the mechanical glove [69].

In addition, intelligent gloves are also a new tool to assist in industrial production and operation. The built-in sensor in the glove can quickly receive and read the movement intensity and position changes of the workers’ fingers, and then, affects the work strength through the action force. For example, General Motors and NASA jointly developed a mechanical glove, the Robo-Glove, with pressure sensors and other sensors, which helped NASA send the space robot Robonaut 2 into outer space in 2011 (Figure 4). The glove consists of five sensors and brakes that mimic the stretching movements of human arm muscles. That leads to less torque when the users accomplish the same amount of work. There is a pressure sensor on the fingers of the glove. When the wearer makes a grasping action of the hand, the pressure sensor will intelligently identify the pressure and do the same action as the hand, saving the strength needed to hold things. The function of the sensors in gloves is similar to human nerves, muscles and tendons, which can be combined to be as dexterous as human hands and have great power at the same time. This ground-breaking muscle imitation technology developed in the field of smart clothing can provide a stronger grip for people in the medical and manufacturing industries.

#### 3.2.1. The 3D Printing in Clothing of Functional Devices

**Figure 5:**
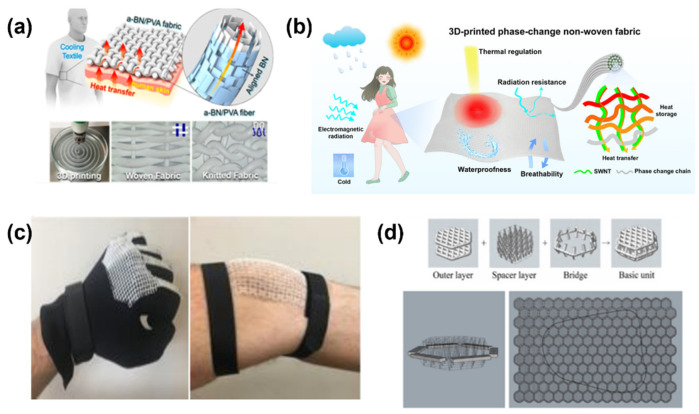
(**a**) The 3D printing of heat-adjusted textiles; (**b**) 3D printing of multifunctional, flexible phase-change nonwovens; (**c**) flexible grids which speed up finger recovery when clenching fists; (**d**) spaced hexagonal grids for fall-protective clothing [72,73,74,75,76].

With the development of 3D printing technology, researchers have achieved certain progress by creating special textile structures or adding functional materials to achieve the 3D printing of functional clothing [70,71]. Using boron nitride (BN) and polyvinyl alcohol (PVA), Gao [72] prepared a type of fiber composite which can be used for 3D printing. This kind of fiber has high mechanical properties and good thermal dispersion, which can realize the thermal adjustment function of 3D-printed clothing (Figure 5a). Yang [73] constructed a PCNF (phosphorylated cellulose fiber) textile fabric based on FDM technology. The uniformly dispersed SWNTs (single-walled carbon nanotubes) in the PCNF form a network structure which can effectively collect heat and quickly transfer it to the solid–solid phase change chain for heat storage. Meanwhile, it is easy to absorb and reflect electromagnetic waves to achieve electromagnetic shielding, which leads to excellent thermal adjustment and radiation resistance of the 3D-printed PCNF fabric clothing (Figure 5b). Pattinson [74] designed a new type of flexible mesh fabric that was printed using thermoplastics. The flexibility can be adjusted by changing the wave structure of the mesh fabric. The flexible mesh fabric can imitate the soft tissue parts of the human body, which have been used in the fist and knee parts (Figure 5c). Wang [75] printed a structural fabric with adjustable bending properties by SLS. The fabric is composed of three-dimensional particles arranged in a layered chain, which can be bent freely in the soft state. However, in the pressurized state, it is more than 25 times harder than that in the soft relaxed state and can withstand more than 30 times the mechanical load. In addition, the fabric has good shape reconfiguration and potential applications in the fields of wearable exoskeletons, tactile architecture and reconfigurable medical support. Park [76] developed clothing that protects against falls by creating hexagonal grids with spaced structures (Figure 5d) via 3D printing technology. Compared with the case without wearing protective clothing, this protective clothing reduces the impact by about 80%. The uniform and repetitive hexagonal grid element developed meets the requirements of flexibility in structure, which is suitable for daily use. The proposed optimized modeling method in this study provides the possibility to print complex and fine, functional clothing using the 3D printing process.

#### 3.2.2. The 3D Printing in Clothing of Electronic Textiles

**Figure 6:**
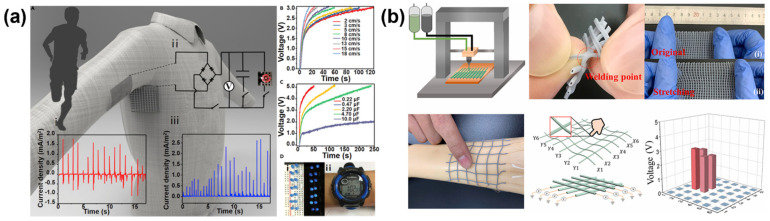
(**a**) Application of the 3D-Printed E-Textile for Energy Management (**A**) Schematic illustration showing smart clothes for energy management and its performance. Inset (**i**) the output *I*_sc_ density of a smart gridline pattern printed on the underarm sleeve of a shirt generated by an arm moving. Inset (**ii**) the rectifying circuit diagram of the power system. Inset (**iii**) the rectified output *I*_sc_ density of the smart pattern; (**B**) Charging curves of a capacitor (3.3 μF) charged using the smart pattern displaced at different speeds; (**C**) Charging curves of different capacitances charged using the smart pattern with a displacement speed of 13 cm/s; (**D**) Photographs showing (**i**) LEDs and (**ii**) an electrical watch driven by the power generated by the 3D-printed E-textile. (**b**) stretchable elastic fibers for tactile sensor of electronic skin. Inset (**i**) original, (**ii**) Stretching. [84,85].

Electronic textiles show great potential in energy collection, health monitoring, human–computer interactions, artificial intelligence and so on [77,78,79,80]. The combination of electronic textiles with 3D printing technology means the accurate and quick printing of complex structures [81,82,83]. Zhang [84] printed core-sheath fiber-based intelligent patterns on clothing textiles using 3D printing technology. These patterns are composed of core-sheath fibers, of which carbon nanotubes (CNTs) are the core fibers and silk fibroin (SF) is the sheath fibers. These fibers can obtain biological energy from the movement of the human body. Then, the collected energy is stored in capacitors which help to make a variety of personalized integrated electronic textiles (Figure 6a). Chen [85] prepared stretchable elastic fibers with a coaxial core-sheath structure by 3D printing technology. PTFE particles and graphene as fillers can accurately respond to the contact position and pressure, which can realize the function of tactile sensors. This 3D printing manufacturing method avoids the complex weaving process of traditional fabrics and can be customized and produced on a large scale. In addition, this 3D-printed product has excellent tensile properties, good structural and functional stability, and convenient washing resistance, which makes it promising for use in wearable electronic products (Figure 6b).

#### 3.2.3. The 3D Printing in Clothing of Exoskeleton Wearing Devices

The advantage of 3D printing is that it can be customized easily, which has great application prospects in orthopedic implants [86], auxiliary preoperative simulations [87], biological printing organs [88] and other applications in medical fields. In addition, 3D printing is also used in the manufacture of personalized medical devices, which makes the wearable medical devices have the best shape, size and mechanical properties [89,90,91,92].

**Figure 7:**
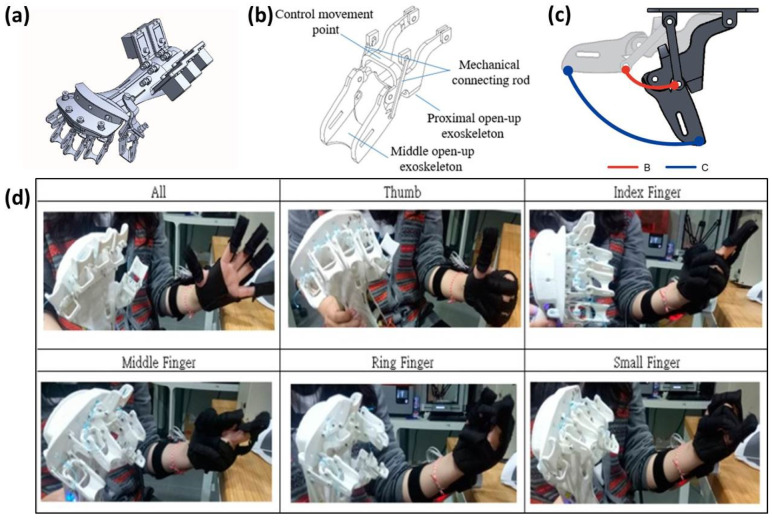
Design and development of a wearable exoskeleton system for stroke rehabilitation. (**a**) and (**b**) are schematics of the exoskeleton; (**c**) schematic of simulated finger joint movement arc; (**d**) actual movements of each finger joint [93].

Ou [93] designed a hand rehabilitation exoskeleton for stroke patients, as shown in Figure 7, which can help patients straighten their fingers and open their palms to simulate spasm rehabilitation training. The device can assist the metacarpophalangeal joint and proximal interphalangeal joint of each finger to bend 0–70° and 0–90°, respectively. The material of the prototype made through 3D printing is polylactic acid and the total cost is much lower than the market price. In the model design stage, the individual’s rehabilitation needs can be met according to the degree of illness, palm width and finger length. In addition, patients can choose to carry out rehabilitation training in their own home, which will improve their function and quality of life.

**Figure 8:**
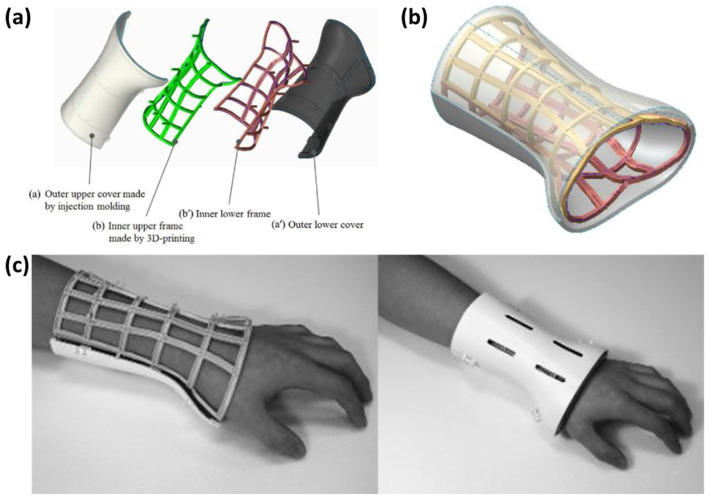
Hand rehabilitation exoskeleton. (**a**) Main concept of the hybrid model for wrist orthosis. (**b**) Combination of the upper cover and inner frame. (**c**) Wearing the new cast [94].

Kim [94] studied a hybrid manufacturing method for wrist orthosis, which uses three-dimensional printing and injection molding technology to make the inner and outer layers of hand protectors, respectively, as shown in Figure 8. At present, the plaster used for fracture treatment makes the arms immovable and not ventilated. Thus, patients wearing plaster for a long time can easily obtain skin diseases and inflammatory infections. The core concept of hybrid manufacturing is to divide the protective gear into two parts: the internal structure of the wrapped skin and the shell fixed on the internal structure. The inner arm model is collected by 3D scanning and the frame that fits to the human body is printed after processing, whereas the outer side is pre-molded according to the size of the wrist to reduce the impact on the fracture. Then, the two parts are fixed to each other through the reserved buckle. It has been proved that the new wrist orthosis has the characteristics of being moderate strength and light weight, and having good ventilation. Besides, the production-time cost is 2/3 times shorter than that of the previous whole printing method.

**Figure 9:**
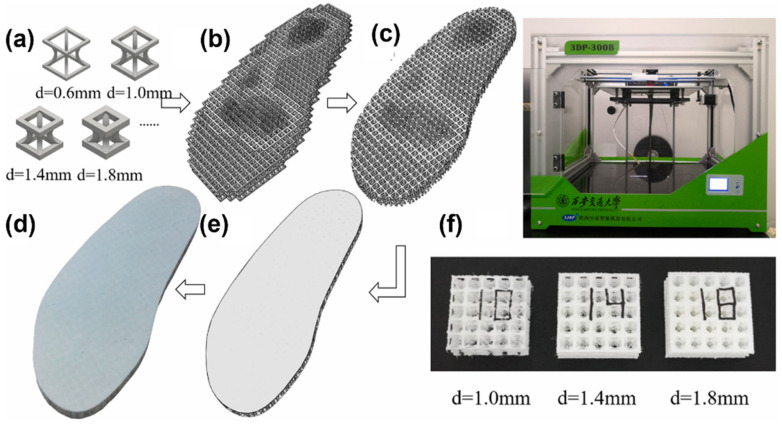
The process of designing and manufacturing gradient structure insole. (**a**) Different porous units to assembled (**b**) Porous substrate (**c**) Boolean intersection of the porous substrate and the original insole (**d**) The complete customized porous insole model (**e**) Manufactured porous customized flat insole and full contact insole (**f**) The printing machine and the 3D-printed testing samples [95].

TangLei [95] used gradient structure characteristics to optimize the stress distribution of the interface between sole and insole. The plantar pressure is simulated by the finite element analysis method, which lays a foundation for the stiffness adjustment in the process of optimization. The soft structure is used in the area with high plantar pressure, whereas the hard structure is used in the area with low plantar pressure. The stiffness, here, is related to the design parameters of porous elements. As shown in Figure 9, the insole is made using FDM technology and the plantar pressure is measured on a gait analyzer. The results show that the optimized insole increases the foot contact area by about 30% and reduces the peak contact pressure by 35%. It is proved that the gradient design method can be used to customize personalized insoles to provide a better decompression effect.

## 4. Challenges and Prospects

Although great progress has been made in 3D printing in the production of clothing, footwear and jewelry accessories, the research on 3D printing in the clothing field still lags behind other fields due to the coupling of printing technology and printing raw materials. For example, the 3D-printed products from common ABS, PLA and other traditional materials are mostly at the level of conceptual clothing, as comfort is difficult to match with traditional clothing. With the development and maturity of 3D printing technology, its application in the smart clothing field can provide personalized customization and make clothing with highly complex structures. For functional clothing and electronic textiles, their fabric special structures are achieved by optimizing printing parameters during the 3D printing process. However, the printing of raw materials and their structure is still the biggest limitation for the development of 3D printing in the clothing field. Besides, the scientific issues on the correlation between material structure and properties of 3D-printed products deepened further research. Therefore, the development of 3D printing in the field of clothing will continue be to explored to find the printing process suitable for the corresponding materials’ structure, which will also be the focus of our further work. It can be predicted that the rise in smart materials and the updates in 3D printing technology will promote the development of 3D printing in the field of smart clothing.

## Data Availability

The data that support the findings of this study are available from the corresponding authors, G.M. and Z.Z., upon reasonable request.
